# Supplementary material for: Metabolic Signatures in Coronary Artery Disease: Results from the BioHEART-CT Study
Source: Cells. 2021 Apr 22;10(5):980. doi: 10.3390/cells10050980 (PMC8145337; doi:10.3390/cells10050980)
Supplement: Supplementary file 1 [file cells-10-00980-s001.zip › cells-1163049-supplementary.pdf]

Supplementary Table 1: Metabolic pathways

Metabolite	Super-Pathway	Sub-Pathway
**α-keto-ß-methylvaleric acid 1**	Amino Acid	Leucine, Isoleucine and Valine Metabolism
**α-keto-ß-methylvaleric acid 2**	Amino Acid	Leucine, Isoleucine and Valine Metabolism
**1-methylhistamine**	Amino Acid	Histidine Metabolism
**2-Arachidonyl glycerol**	Lipid	Glycerolipid Metabolism
**2'-deoxyadenosine**	Nucleotide	Pyrimidine Metabolism, Cytidine containing
**3-IPA**	Amino Acid	Tryptophan Metabolism
**3HK**	Amino Acid	Tryptophan Metabolism
**5-Aminolevulinic Acid**	Energy	Heme synthesis
**Acetylcarnitine**	Lipid	Fatty Acid Metabolism(Acyl Carnitine)
**Adenosine**	Nucleotide	Purine Metabolism, Adenine containing
**Alanine**	Amino Acid	Alanine and Aspartate Metabolism
**Anandamide**	Lipid	Endocannabinoid
**Arachidonic acid**	Lipid	Fatty acid Metabolism
**Arginine**	Amino Acid	Urea cycle; Arginine and Proline Metabolism
**Asparagine**	Amino Acid	Alanine and Aspartate Metabolism
**Aspartate**	Amino Acid	Alanine and Aspartate Metabolism
**Betaine**	Amino Acid	Glycine, Serine and Threonine Metabolism
**Butyrylcarnitine**	Lipid	Fatty Acid Metabolism
**cAMP**	Nucleotide	Purine Metabolism, Adenine containing
**Carnitine**	Lipid	Carnitine Metabolism
**Choline**	Lipid	Phospholipid Metabolism
**Creatine**	Amino Acid	Creatine Metabolism
**Cysteamine**	Amino Acid	Methionine, Cysteine, SAM and Taurine Metabolism
**Cysteine**	Amino Acid	Methionine, Cysteine, SAM and Taurine Metabolism
**Cytosine**	Nucleotide	Pyrimidine Metabolism, Cytidine containing
**DMGV**	amino Acid	Urea cycle; Arginine and Proline Metabolism
**GlucosePos2**	Carbohydrate	Glycolysis, Gluconeogenesis, and Pyruvate Metabolism
**Glutamate**	Amino Acid	Glutamate Metabolism
**Glutamine**	Amino Acid	Glutamate Metabolism
**Histidine**	Amino Acid	Histidine Metabolism
**Isoleucine_Leucine**	Amino Acid	Leucine, Isoleucine and Valine Metabolism
**Kynurenic acid**	Amino Acid	Tryptophan Metabolism

**L-Homoserine**	Amino Acid	Glycine, Serine and Threonine Metabolism
**Methionine**	Amino Acid	Methionine, Cysteine, SAM and Taurine Metabolism
**Phenylalanine**	Amino Acid	Phenylalanine
**Phosphocholine**	Lipid	Phosphatidylcholine
**Proline**	Amino Acid	Proline
**Riboflavin**	Cofactors and Vitamins	Riboflavin
**Serine**	Amino Acid	Glycine, Serine and Threonine Metabolism
**Serotonin**	Amino Acid	Tryptophan Metabolism
**Spermine**	Amino Acid	Polyamine Metabolism
**Taurine**	Amino Acid	Taurine
**Thiamine**	Carbohydrate	Glycolysis, Gluconeogenesis, and Pyruvate Metabolism
**Threonine**	Amino Acid	Threonine
**Thymidine**	Nucleotide	Pyrimidine Metabolism, Thymine containing
**TMAO**	Lipid	Phospholipid Metabolism
**trans-HYP**	Amino Acid	Urea cycle; Arginine and Proline Metabolism
**Tyrosine**	Amino Acid	Tyrosine Metabolism
**Uridine**	Nucleotide	Pyrimidine Metabolism, Uracil containing
**Valine**	Amino Acid	Leucine, Isoleucine and Valine Metabolism
**Valine-d8**	Amino Acid	Leucine, Isoleucine and Valine Metabolism

Supplementary Table 2. Pearson correlations of DMGV with traditional CAD risk factors.

	**Pearson Correlation**	**P value**
Hypertension	0.145	**<0.001**
Hypercholesterolaemia	0.029	0.359
Diabetes mellitus	0.172	<**0.001**
Smoking	0.078	**0.014**
Body mass index	0.218	**<0.001**
Age	0.135	**<0.001**
Gender	0.011	0.729

Supplementary Table 3. DMGV associations with presence of CAD, adjusted for traditional risk factors (one at a time).

	**CAD**			
	**OR**	**Lower CI**	**Upper CI**	**P value**
**DMGV (unadjusted)**	1.41	1.12	1.79	**0.004**
**DMGV adjusted for:**				
Hypertension	1.30	1.02	1.66	**0.033**
Hypercholesterolaemia	1.40	1.10	1.79	**0.006**
Diabetes mellitus	1.37	1.08	1.74	**0.011**
Smoking	1.42	1.12	1.80	**0.004**
Body mass index	1.02	0.99	1.05	0.258
Age	1.15	0.88	1.50	0.322
Gender	1.41	1.11	1.80	**0.005**

Supplementary Table 4. Univariate Binary Logistic Regression Associations with Presence of CAD.

Metabolite	Odds Ratio	lower_CI	upper_CI	P value	FDR Adjusted P-value
α-keto-ß-methylvaleric acid 1	0.82	0.53	1.26	0.357	0.612
α-keto-ß-methylvaleric acid 2	1.23	0.91	1.67	0.184	0.424
Acetylcarnitine	0.86	0.70	1.06	0.166	0.399
Adenosine	1.47	1.04	2.08	0.031	0.207
Alanine	0.97	0.78	1.22	0.808	0.948
Anandamide	0.88	0.62	1.25	0.464	0.723
Arachidonic.acid	0.72	0.48	1.07	0.104	0.324
Arginine	1.00	0.75	1.32	0.972	0.972
Asparagine	1.60	1.13	2.27	0.008	0.089
Aspartate	1.03	0.76	1.39	0.846	0.948
Betaine	0.95	0.79	1.15	0.621	0.850
Butyrylcarnitine	1.02	0.79	1.32	0.868	0.948
cAMP	1.39	1.12	1.74	0.003	0.075
Carnitine	1.14	0.78	1.65	0.498	0.755
Choline	1.45	0.99	2.13	0.057	0.233
Colchicine	0.82	0.64	1.06	0.134	0.375
Creatine	0.97	0.68	1.38	0.844	0.948
Cysteamine	0.76	0.54	1.08	0.122	0.359
Cysteine	1.04	0.70	1.54	0.844	0.948
Cytosine	1.08	0.79	1.46	0.642	0.850
DMGV	1.41	1.12	1.79	0.004	0.075
GlucosePos2	0.99	0.68	1.44	0.946	0.972
Glutamate	1.15	0.84	1.57	0.398	0.645
Glutamine	0.94	0.64	1.37	0.734	0.948
Histidine	0.79	0.50	1.24	0.299	0.589
Isoleucine_Leucine	0.96	0.70	1.31	0.796	0.948
Kynurenic acid	1.22	1.00	1.48	0.048	0.233
L-Homoserine	1.07	0.81	1.40	0.631	0.850
Methionine	1.03	0.75	1.41	0.877	0.948
Phenylalanine	0.99	0.65	1.48	0.943	0.972
Phosphocholine	1.16	0.82	1.64	0.401	0.645
Proline	1.58	1.13	2.21	0.008	0.089
Riboflavin	1.15	0.97	1.35	0.101	0.324
Serine	0.91	0.74	1.12	0.358	0.612
Serotonin	0.89	0.59	1.33	0.557	0.804

**Spermine**	1.43	1.08	1.90	0.013	0.116
**Taurine**	0.93	0.71	1.20	0.561	0.804
**Thiamine**	1.36	1.01	1.84	0.042	0.223
**Threonine**	0.82	0.56	1.20	0.311	0.589
**Thymidine**	0.58	0.35	0.97	0.037	0.216
**TMAO**	0.85	0.62	1.16	0.305	0.589
**trans-HYP**	0.84	0.69	1.02	0.084	0.318
**Tyrosine**	0.71	0.51	1.01	0.054	0.233
**Uridine**	0.64	0.47	0.86	0.004	0.075
**Valine**	1.20	0.86	1.69	0.281	0.589
**Valine-d8**	0.99	0.78	1.28	0.963	0.972
**1-methylhistamine**	1.25	0.83	1.86	0.285	0.589
**2’-deoxyadenosine**	0.90	0.73	1.11	0.333	0.608
**2-Arachidonyl glycerol**	0.65	0.45	0.95	0.026	0.200
**3-deaazadenosine**	0.76	0.51	1.12	0.165	0.399
**3-IPA**	0.90	0.79	1.02	0.104	0.324
**3HK**	1.14	0.95	1.35	0.153	0.399
**5-Aminolevulinic Acid**	0.98	0.83	1.16	0.792	0.948

Supplementary Table 5. Univariate Binary Logistic Regression Associations with Presence of Calcified Plaque.

Metabolite	Odds Ratio	lower _CI	upper_CI	P value	FDR Adjusted P-value
α-keto-ß-methylvaleric acid 1	0.89	0.59	1.34	0.568	0.739
α-keto-ß-methylvaleric acid 2	1.18	0.88	1.58	0.268	0.617
Acetylcarnitine	0.89	0.73	1.09	0.258	0.617
Adenosine	1.47	1.05	2.05	0.024	0.130
Alanine	0.99	0.79	1.22	0.894	0.894
Anandamide	0.85	0.61	1.19	0.345	0.651
Arachidonic acid	0.69	0.47	1.02	0.063	0.257
Arginine	1.11	0.85	1.46	0.438	0.683
Asparagine	1.62	1.15	2.27	0.006	0.044
Aspartate	1.09	0.81	1.45	0.576	0.739
Betaine	0.96	0.80	1.15	0.653	0.786
Butyrylcarnitine	1.11	0.87	1.42	0.414	0.683
cAMP	1.36	1.11	1.67	0.003	0.031
Carnitine	1.16	0.81	1.67	0.421	0.683
Choline	1.32	0.91	1.92	0.140	0.469
Colchicine	0.83	0.64	1.07	0.156	0.487
Creatine	0.95	0.67	1.34	0.765	0.834
Cysteamine	0.71	0.51	1.00	0.050	0.219
Cysteine	1.22	0.83	1.78	0.307	0.625
Cytosine	1.14	0.84	1.53	0.402	0.683
DMGV	1.59	1.26	2.01	0.0001	0.006
GlucosePos2	0.87	0.61	1.26	0.464	0.703
Glutamate	1.21	0.89	1.65	0.214	0.539
Glutamine	0.90	0.62	1.30	0.561	0.739
Histidine	0.91	0.59	1.42	0.690	0.796
Isoleucine_Leucine	0.92	0.68	1.25	0.604	0.745
Kynurenic acid	1.32	1.09	1.60	0.004	0.037
L-Homoserine	1.15	0.89	1.50	0.291	0.625
Methionine	1.10	0.81	1.49	0.554	0.739
Phenylalanine	1.12	0.75	1.67	0.570	0.739
Phosphocholine	1.03	0.74	1.43	0.879	0.894
Proline	1.69	1.21	2.36	0.002	0.030
Riboflavin	1.14	0.98	1.33	0.100	0.380
Serine	0.97	0.80	1.19	0.771	0.834

**Serotonin**	0.81	0.55	1.20	0.300	0.625
**Spermine**	1.54	1.17	2.02	0.002	0.030
**Taurine**	0.93	0.72	1.20	0.585	0.739
**Thiamine**	1.36	1.02	1.81	0.035	0.170
**Threonine**	0.96	0.67	1.38	0.823	0.872
**Thymidine**	0.53	0.32	0.87	0.012	0.072
**TMAO**	0.98	0.72	1.32	0.885	0.894
**trans-HYP**	0.87	0.72	1.05	0.142	0.469
**Tyrosine**	0.81	0.58	1.11	0.191	0.539
**Uridine**	0.63	0.47	0.84	0.002	0.030
**Valine**	1.17	0.84	1.61	0.356	0.651
**Valine.d8**	0.96	0.75	1.22	0.711	0.802
**1-methylhistamine**	1.12	0.76	1.66	0.556	0.739
**2’-deoxyadenosine**	0.92	0.75	1.13	0.431	0.683
**2-Arachidonyl glycerol**	0.62	0.43	0.89	0.010	0.066
**3-deaazadenosine**	0.82	0.56	1.21	0.318	0.625
**3-IPA**	0.92	0.82	1.05	0.207	0.539
**3HK**	1.12	0.94	1.32	0.198	0.539
**5-Aminolevulinic Acid**	0.97	0.82	1.14	0.691	0.796

Supplementary Table 6. Univariate Binary Logistic Regression Associations with Presence of Non-calcified Plaque.

Metabolite	Odds Ratio	lower _CI	upper_CI	P value	FDR Adjusted P-value
**α-keto-ß-methylvaleric acid 1**	1.02	0.68	1.52	0.941	0.985
**α-keto-ß-methylvaleric acid 2**	1.01	0.76	1.35	0.932	0.985
**Acetylcarnitine**	0.94	0.77	1.13	0.496	0.822
**Adenosine**	1.18	0.86	1.63	0.294	0.653
**Alanine**	1.01	0.82	1.25	0.923	0.985
**Anandamide**	0.85	0.61	1.19	0.340	0.721
**Arachidonic acid**	0.67	0.45	0.98	0.040	0.193
**Arginine**	0.89	0.68	1.16	0.394	0.746
**Asparagine**	1.36	0.98	1.90	0.067	0.235
**Aspartate**	0.88	0.66	1.17	0.384	0.746
**Betaine**	0.99	0.83	1.19	0.948	0.985
**Butyrylcarnitine**	1.03	0.81	1.31	0.824	0.930
**cAMP**	1.28	1.06	1.54	0.010	0.129
**Carnitine**	1.29	0.90	1.86	0.169	0.473
**Choline**	1.63	1.12	2.36	0.011	0.129
**Colchicine**	0.86	0.66	1.11	0.235	0.567
**Creatine**	0.96	0.68	1.34	0.809	0.930
**Cysteamine**	0.75	0.54	1.05	0.092	0.271
**Cysteine**	0.82	0.57	1.19	0.295	0.653
**Cytosine**	0.91	0.68	1.22	0.536	0.835
**DMGV**	1.40	1.11	1.75	0.004	0.129
**GlucosePos2**	1.10	0.77	1.58	0.590	0.845
**Glutamate**	1.48	1.09	2.01	0.011	0.129
**Glutamine**	0.99	0.69	1.43	0.973	0.991
**Histidine**	0.62	0.40	0.96	0.031	0.166
**Isoleucine_Leucine**	1.08	0.80	1.45	0.620	0.865
**Kynurenic acid**	1.18	0.98	1.41	0.088	0.271
**L-Homoserine**	1.00	0.77	1.29	0.999	0.999
**Methionine**	1.21	0.89	1.64	0.220	0.556
**Phenylalanine**	0.88	0.60	1.30	0.526	0.835
**Phosphocholine**	1.15	0.83	1.59	0.415	0.754
**Proline**	1.38	1.00	1.90	0.049	0.198
**Riboflavin**	1.15	0.98	1.34	0.083	0.271
**Serine**	1.03	0.84	1.25	0.786	0.930
**Serotonin**	0.78	0.53	1.15	0.215	0.556

**Spermine**	1.36	1.04	1.79	0.025	0.162
**Taurine**	0.90	0.70	1.16	0.427	0.754
**Thiamine**	1.31	1.00	1.73	0.053	0.201
**Threonine**	1.10	0.77	1.58	0.588	0.845
**Thymidine**	0.57	0.35	0.92	0.022	0.162
**TMAO**	0.72	0.53	0.97	0.029	0.166
**trans-HYP**	0.94	0.78	1.13	0.489	0.822
**Tyrosine**	0.93	0.68	1.27	0.641	0.872
**Uridine**	0.69	0.52	0.92	0.012	0.129
**Valine**	1.06	0.77	1.45	0.744	0.930
**Valine.d8**	1.05	0.83	1.34	0.668	0.885
**1-methylhistamine**	1.08	0.74	1.58	0.697	0.900
**2’-deoxyadenosine**	1.03	0.84	1.25	0.779	0.930
**2-Arachidonyl glycerol**	0.90	0.63	1.28	0.554	0.839
**3-deaazadenosine**	1.05	0.72	1.52	0.819	0.930
**3-IPA**	0.86	0.76	0.97	0.017	0.155
**3HK**	1.19	1.00	1.40	0.047	0.198
**5-Aminolevulinic Acid**	1.08	0.92	1.26	0.365	0.743

Supplementary Table 7. Univariate Binary Logistic Regression Associations with Presence of Obstructive CAD.

Metabolite	Odds Ratio	lower_CI	upper_CI	P value	FDR Adjusted P-value
α-keto-ß-methylvaleric acid 1	0.9	0.45	1.78	0.763	0.833
α-keto-ß-methylvaleric acid 2	1.11	0.72	1.71	0.644	0.827
Acetylcarnitine	0.86	0.65	1.14	0.295	0.54
Adenosine	1.74	1.09	2.78	0.020	0.128
Alanine	1.02	0.74	1.42	0.903	0.921
Anandamide	0.81	0.5	1.31	0.39	0.607
Arachidonic acid	0.51	0.27	0.95	0.034	0.139
Arginine	1.01	0.68	1.5	0.966	0.966
Asparagine	1.89	1.12	3.17	0.016	0.128
Aspartate	1.18	0.78	1.78	0.429	0.626
Betaine	1.05	0.8	1.38	0.711	0.833
Butyrylcarnitine	1.18	0.81	1.72	0.385	0.607
cAMP	1.39	1.06	1.82	0.017	0.128
Carnitine	1.36	0.75	2.47	0.31	0.54
Choline	1.24	0.72	2.16	0.439	0.626
Colchicine	0.70	0.43	1.13	0.141	0.394
Creatine	1.12	0.66	1.9	0.671	0.827
Cysteamine	0.54	0.31	0.92	0.024	0.128
Cysteine	1.06	0.62	1.81	0.843	0.894
Cytosine	1.29	0.81	2.06	0.284	0.54
DMGV	2.33	1.59	3.43	<0.001	<0.001
GlucosePos2	1.12	0.67	1.87	0.669	0.827
Glutamate	1.25	0.78	2.01	0.361	0.597
Glutamine	1.1	0.61	1.98	0.75	0.833
Histidine	0.63	0.33	1.19	0.153	0.405
Isoleucine_Leucine	0.93	0.57	1.5	0.752	0.833
Kynurenic acid	1.49	1.1	2.02	0.01	0.128
L-Homoserine	1.09	0.75	1.6	0.653	0.827
Methionine	1.28	0.81	2.02	0.296	0.54
Phenylalanine	1.84	1.01	3.33	0.046	0.173
Phosphocholine	0.09	0.66	1.81	0.731	0.833
Proline	1.99	1.2	3.28	0.007	0.128
Riboflavin	1.34	1.06	1.67	0.012	0.128
Serine	0.98	0.73	1.32	0.889	0.921

**Serotonin**	0.66	0.38	1.13	0.129	0.38
**Spermine**	1.56	1.04	2.35	0.0321	0.139
**Taurine**	0.79	0.54	1.14	0.202	0.511
**Thiamine**	1.5	1.04	2.17	0.0303	0.139
**Threonine**	0.76	0.46	1.27	0.296	0.54
**Thymidine**	0.51	0.24	1.08	0.0807	0.267
**TMAO**	0.79	0.52	1.22	0.292	0.54
**trans-HYP**	0.86	0.67	1.12	0.259	0.54
**Tyrosine**	0.69	0.43	1.11	0.128	0.38
**Uridine**	0.6	0.4	0.9	0.0125	0.128
**Valine**	1.33	0.8	2.13	0.23	0.53
**Valine-d8**	1.16	0.8	1.68	0.449	0.626
**1-methylhistamine**	0.78	0.43	1.4	0.408	0.618
**2’-deoxyadenosine**	0.86	0.65	1.15	0.316	0.54
**2-Arachidonyl glycerol**	0.56	0.33	0.93	0.024	0.128
**3-deaazadenosine**	0.84	0.48	1.47	0.534	0.726
**3-IPA**	0.85	0.71	1.02	0.077	0.267
**3HK**	1.16	0.91	1.47	0.223	0.53
**5-Aminolevulinic Acid**	0.97	0.77	1.22	0.77	0.833

Supplementary Table 8. Univariate Linear Regression Associations with Gensini Score.

Metabolite	Beta	lower_ CI.2.5 %	upper_ CI.97.5 %	P value	FDR Adjusted P-value
α-keto-ß-methylvaleric acid 1	-0.06	-0.35	0.23	0.690	0.871
α-keto-ß-methylvaleric acid 2	-0.20	-0.42	0.01	0.063	0.418
Acetylcarnitine	0.02	-0.12	0.17	0.781	0.905
Adenosine	0.10	-0.13	0.33	0.388	0.655
Alanine	-0.05	-0.21	0.11	0.512	0.776
Anandamide	-0.06	-0.32	0.19	0.628	0.833
Arachidonic acid	-0.14	-0.43	0.14	0.321	0.655
Arginine	0.02	-0.19	0.22	0.865	0.954
Asparagine	0.01	-0.24	0.25	0.965	0.985
Aspartate	0.00	-0.22	0.22	0.993	0.993
Betaine	0.04	-0.10	0.18	0.561	0.827
Butyrylcarnitine	0.09	-0.10	0.27	0.365	0.655
cAMP	0.06	-0.07	0.18	0.357	0.655
Carnitine	0.29	0.03	0.54	0.029	0.219
Choline	-0.04	-0.32	0.24	0.785	0.905
Colchicine	0.04	-0.17	0.25	0.720	0.887
Creatine	0.02	-0.23	0.26	0.892	0.954
Cysteamine	-0.18	-0.41	0.06	0.145	0.548
Cysteine	-0.03	-0.31	0.25	0.833	0.939
Cytosine	0.11	-0.10	0.33	0.309	0.655
DMGV	0.30	0.14	0.45	0.0002	0.014
GlucosePos2	0.14	-0.15	0.44	0.339	0.655
Glutamate	0.14	-0.09	0.36	0.234	0.655
Glutamine	0.13	-0.14	0.39	0.349	0.655
Histidine	-0.19	-0.52	0.13	0.247	0.655
Isoleucine_Leucine	-0.06	-0.28	0.15	0.579	0.830
Kynurenic acid	0.17	0.04	0.30	0.012	0.205
L-Homoserine	-0.01	-0.21	0.18	0.900	0.954
Methionine	0.27	0.04	0.50	0.020	0.218
Phenylalanine	0.33	0.04	0.62	0.025	0.218
Phosphocholine	-0.12	-0.36	0.12	0.331	0.655
Proline	0.18	-0.06	0.41	0.136	0.548
Riboflavin	0.15	0.04	0.26	0.009	0.205
Serine	0.04	-0.11	0.19	0.616	0.833
Serotonin	-0.16	-0.45	0.13	0.278	0.655
Spermine	0.08	-0.12	0.28	0.427	0.685
Taurine	0.06	-0.12	0.25	0.497	0.775

**Thiamine**	0.17	-0.03	0.37	0.096	0.509
**Threonine**	0.13	-0.15	0.41	0.371	0.655
**Thymidine**	-0.39	-0.73	-0.05	0.023	0.218
**TMAO**	-0.16	-0.39	0.07	0.185	0.653
**trans-HYP**	0.02	-0.12	0.16	0.741	0.893
**Tyrosine**	0.11	-0.13	0.34	0.379	0.655
**Uridine**	-0.17	-0.40	0.05	0.123	0.548
**Valine**	-0.11	-0.35	0.13	0.364	0.655
**Valine-d8**	0.05	-0.13	0.22	0.607	0.833
**1-methylhistamine**	-0.15	-0.44	0.13	0.293	0.655
**2’-deoxyadenosine**	0.06	-0.08	0.21	0.395	0.655
**2-Arachidonyl.glycerol**	-0.01	-0.28	0.27	0.966	0.985
**3-deaazadenosine**	0.26	-0.02	0.54	0.073	0.429
**3-IPA**	-0.07	-0.17	0.02	0.137	0.548
**3HK**	0.07	-0.06	0.20	0.263	0.655
**5-Aminolevulinic Acid**	0.03	-0.09	0.14	0.675	0.871

Supplementary Table 9. Univariate Linear Regression Associations with CACS.

Metabolite	Beta	lower_CI .2.5 %	upper_CI .97.5 %	P value	FDR Adjusted P-value
**α-keto-ß-methylvaleric acid 1**	-0.42	-1.10	0.26	0.223	0.657
**α-keto-ß-methylvaleric acid 2**	-0.44	-0.93	0.05	0.079	0.464
**Acetylcarnitine**	0.03	-0.31	0.37	0.862	0.971
**Adenosine**	0.03	-0.50	0.56	0.912	0.971
**Alanine**	-0.16	-0.52	0.20	0.390	0.784
**Anandamide**	0.14	-0.46	0.74	0.644	0.892
**Arachidonic acid**	-0.15	-0.79	0.50	0.657	0.892
**Arginine**	0.05	-0.44	0.53	0.852	0.971
**Asparagine**	-0.16	-0.73	0.41	0.573	0.882
**Aspartate**	0.09	-0.42	0.61	0.721	0.932
**Betaine**	0.14	-0.18	0.46	0.387	0.784
**Butyrylcarnitine**	0.32	-0.12	0.76	0.155	0.600
**cAMP**	0.17	-0.12	0.46	0.240	0.668
**Carnitine**	0.62	0.02	1.22	0.044	0.334
**Choline**	-0.07	-0.73	0.59	0.830	0.971
**Colchicine**	-0.04	-0.50	0.43	0.879	0.971
**Creatine**	0.31	-0.25	0.88	0.273	0.677
**Cysteamine**	-0.41	-0.98	0.15	0.154	0.600
**Cysteine**	-0.45	-1.10	0.20	0.171	0.600
**Cytosine**	-0.01	-0.51	0.49	0.960	0.971
**DMGV**	0.49	0.11	0.87	0.011	0.292
**GlucosePos2**	0.40	-0.29	1.08	0.258	0.677
**Glutamate**	0.05	-0.48	0.58	0.863	0.971
**Glutamine**	0.41	-0.18	1.01	0.174	0.600
**Histidine**	-0.51	-1.27	0.24	0.181	0.600
**Isoleucine_Leucine**	-0.14	-0.63	0.35	0.582	0.882
**Kynurenic acid**	0.31	0.00	0.62	0.050	0.334
**L-Homoserine**	-0.16	-0.61	0.28	0.479	0.859
**Methionine**	0.54	0.01	1.07	0.045	0.334
**Phenylalanine**	0.88	0.23	1.53	0.008	0.292
**Phosphocholine**	-0.30	-0.87	0.27	0.296	0.681
**Proline**	0.56	0.01	1.11	0.045	0.334
**Riboflavin**	0.29	0.04	0.55	0.022	0.334
**Serine**	-0.19	-0.55	0.16	0.281	0.677
**Serotonin**	-0.21	-0.87	0.45	0.541	0.882
**Spermine**	-0.11	-0.58	0.36	0.643	0.892
**Taurine**	0.28	-0.15	0.70	0.197	0.616

**Thiamine**	0.52	0.06	0.98	0.026	0.334
**Threonine**	-0.21	-0.88	0.45	0.529	0.882
**Thymidine**	-0.32	-1.11	0.47	0.430	0.815
**TMAO**	-0.46	-0.99	0.08	0.093	0.464
**trans-HYP**	0.04	-0.28	0.36	0.805	0.971
**Tyrosine**	0.24	-0.30	0.78	0.382	0.784
**Uridine**	-0.02	-0.53	0.49	0.933	0.971
**Valine**	-0.14	-0.70	0.42	0.621	0.892
**Valine-d8**	0.14	-0.26	0.54	0.486	0.859
**1-methylhistamine**	-0.14	-0.81	0.54	0.694	0.919
**2'-deoxyadenosine**	0.10	-0.25	0.44	0.576	0.882
**2-Arachidonyl glycerol**	-0.03	-0.66	0.61	0.938	0.971
**3-deaazadenosine**	0.55	-0.10	1.20	0.096	0.464
**3-IPA**	0.00	-0.22	0.23	0.971	0.971
**3HK**	0.13	-0.17	0.42	0.400	0.784
**5-Aminolevulinic Acid**	-0.02	-0.30	0.26	0.897	0.971

Supplementary Table 10. Univariate Linear Regression Associations with Soft Plaque Score.

Metabolite	Beta	lower_CI. 2.5 %	upper_CI .97.5 %	P value	FDR Adjusted P-value
**α-keto-ß-methylvaleric acid 1**	-0.02	-0.30	0.27	0.900	0.946
**α-keto-ß-methylvaleric acid 2**	-0.01	-0.24	0.21	0.901	0.946
**Acetylcarnitine**	-0.02	-0.18	0.13	0.766	0.946
**Adenosine**	0.12	-0.12	0.35	0.327	0.946
**Alanine**	-0.01	-0.18	0.15	0.891	0.946
**Anandamide**	-0.08	-0.35	0.19	0.552	0.946
**Arachidonic acid**	-0.03	-0.33	0.27	0.828	0.946
**Arginine**	-0.03	-0.24	0.19	0.818	0.946
**Asparagine**	-0.09	-0.34	0.16	0.490	0.946
**Aspartate**	-0.05	-0.28	0.18	0.684	0.946
**Betaine**	0.07	-0.07	0.21	0.329	0.946
**Butyrylcarnitine**	0.08	-0.12	0.27	0.437	0.946
**cAMP**	-0.03	-0.16	0.10	0.672	0.946
**Carnitine**	0.16	-0.10	0.42	0.229	0.946
**Choline**	-0.29	-0.59	0.00	0.054	0.774
**Colchicine**	-0.02	-0.23	0.18	0.827	0.946
**Creatine**	-0.06	-0.31	0.19	0.621	0.946
**Cysteamine**	0.05	-0.19	0.30	0.665	0.946
**Cysteine**	0.03	-0.27	0.33	0.847	0.946
**Cytosine**	0.08	-0.15	0.32	0.481	0.946
**DMGV**	0.15	-0.02	0.32	0.089	0.842
**GlucosePos2**	0.11	-0.19	0.41	0.483	0.946
**Glutamate**	0.12	-0.12	0.36	0.326	0.946
**Glutamine**	0.01	-0.25	0.28	0.927	0.946
**Histidine**	-0.13	-0.48	0.22	0.470	0.946

**Isoleucine_Leucine**	0.06	-0.16	0.28	0.612	0.946
**Kynurenic acid**	0.08	-0.06	0.22	0.263	0.946
**L-Homoserine**	-0.04	-0.24	0.16	0.668	0.946
**Methionine**	0.20	-0.04	0.43	0.095	0.842
**Phenylalanine**	0.29	-0.01	0.58	0.058	0.774
**Phosphocholine**	-0.07	-0.31	0.18	0.584	0.946
**Proline**	0.04	-0.20	0.28	0.753	0.946
**Riboflavin**	0.17	0.06	0.28	0.003	0.176
**Serine**	-0.01	-0.17	0.15	0.890	0.946
**Serotonin**	-0.04	-0.33	0.26	0.798	0.946
**Spermine**	0.07	-0.13	0.28	0.496	0.946
**Taurine**	0.03	-0.16	0.22	0.758	0.946
**Thiamine**	0.23	0.02	0.44	0.028	0.755
**Threonine**	0.04	-0.26	0.34	0.804	0.946
**Thymidine**	-0.26	-0.61	0.08	0.134	0.946
**TMAO**	-0.05	-0.29	0.19	0.707	0.946
**trans-HYP**	0.00	-0.15	0.15	0.984	0.984
**Tyrosine**	0.08	-0.17	0.32	0.539	0.946
**Uridine**	0.02	-0.21	0.25	0.858	0.946
**Valine**	0.03	-0.22	0.28	0.792	0.946
**Valine-d8**	0.05	-0.13	0.23	0.559	0.946
**1-methylhistamine**	0.03	-0.27	0.33	0.840	0.946
**2’-deoxyadenosine**	0.01	-0.15	0.16	0.928	0.946
**2-Arachidonyl glycerol**	-0.07	-0.36	0.22	0.651	0.946
**3-deaazadenosine**	0.21	-0.09	0.51	0.174	0.946
**3-IPA**	-0.04	-0.14	0.06	0.406	0.946
**3HK**	0.04	-0.10	0.17	0.567	0.946
**5-Aminolevulinic Acid**	-0.01	-0.14	0.11	0.819	0.946

Supplementary Table 11. Multivariable Binary Logistic Regression Associations with Presence of CAD.

Metabolite	Odds Ratio	lower_CI	upper_CI	P value	FDR Adjusted P-value
α-keto-ß-methylvaleric acid 1	0.80	0.47	1.36	0.415	0.936
α-keto-ß-methylvaleric acid 2	1.17	0.80	1.71	0.408	0.936
Acetylcarnitine	0.91	0.71	1.16	0.434	0.936
Adenosine	1.19	0.77	1.82	0.437	0.936
Alanine	0.90	0.68	1.19	0.472	0.936
Anandamide	0.91	0.59	1.40	0.678	0.936
Arachidonic acid	1.17	0.72	1.91	0.531	0.936
Arginine	0.98	0.70	1.39	0.919	0.936
Asparagine	1.10	0.71	1.70	0.661	0.936
Aspartate	0.93	0.65	1.34	0.697	0.936
Betaine	0.95	0.75	1.20	0.685	0.936
Butyrylcarnitine	0.87	0.64	1.19	0.380	0.936
cAMP	1.03	0.81	1.30	0.814	0.936
Carnitine	1.02	0.64	1.62	0.936	0.936
Choline	1.47	0.92	2.35	0.104	0.936
Colchicine	0.81	0.57	1.15	0.237	0.936
Creatine	1.05	0.68	1.62	0.839	0.936
Cysteamine	0.98	0.64	1.52	0.934	0.936
Cysteine	1.12	0.69	1.80	0.653	0.936
Cytosine	1.16	0.79	1.69	0.454	0.936
DMGV	0.93	0.69	1.26	0.641	0.936
GlucosePos2	0.88	0.55	1.39	0.580	0.936
Glutamate	0.96	0.65	1.41	0.819	0.936
Glutamine	0.74	0.45	1.23	0.249	0.936
Histidine	0.96	0.54	1.68	0.877	0.936
Isoleucine_Leucine	1.02	0.68	1.51	0.935	0.936
Kynurenic acid	1.07	0.83	1.36	0.616	0.936
L-Homoserine	1.08	0.78	1.51	0.637	0.936
Methionine	0.93	0.63	1.37	0.715	0.936
Phenylalanine	0.85	0.51	1.44	0.556	0.936
Phosphocholine	1.42	0.92	2.20	0.116	0.936
Proline	1.02	0.69	1.52	0.909	0.936
Riboflavin	1.05	0.86	1.29	0.609	0.936
Serine	0.96	0.75	1.24	0.762	0.936
Serotonin	0.93	0.56	1.54	0.768	0.936

**Spermine**	1.21	0.84	1.73	0.309	0.936
**Taurine**	0.99	0.72	1.36	0.934	0.936
**Thiamine**	1.12	0.76	1.63	0.574	0.936
**Threonine**	0.72	0.46	1.14	0.164	0.936
**Thymidine**	1.05	0.57	1.92	0.877	0.936
**TMAO**	0.83	0.57	1.22	0.350	0.936
**trans-HYP**	0.85	0.67	1.07	0.164	0.936
**Tyrosine**	0.84	0.56	1.27	0.414	0.936
**Uridine**	0.69	0.47	1.01	0.057	0.936
**Valine**	1.36	0.89	2.10	0.157	0.936
**Valine-d8**	1.05	0.77	1.42	0.769	0.936
**1-methylhistamine**	1.25	0.76	2.06	0.375	0.936
**2’-deoxyadenosine**	0.97	0.75	1.25	0.816	0.936
**2-Arachidonyl glycerol**	0.87	0.56	1.37	0.554	0.936
**3-deaazadenosine**	0.71	0.44	1.16	0.176	0.936
**3-IPA**	0.86	0.74	1.00	0.053	0.936
**3HK**	1.15	0.92	1.42	0.217	0.936
**5-Aminolevulinic Acid**	0.99	0.80	1.22	0.891	0.936

Supplementary Table 12. Multivariable Binary Logistic Regression Associations with Presence of Calcified CAD.

Metabolite	Odds Ratio	lower_CI	upper_CI	P value	FDR Adjusted P-value
**α-keto-ß-methylvaleric acid 1**	0.94	0.56	1.58	0.805	0.983
**α-keto-ß-methylvaleric acid 2**	1.11	0.77	1.61	0.574	0.983
**Acetylcarnitine**	0.96	0.75	1.23	0.744	0.983
**Adenosine**	1.17	0.77	1.78	0.461	0.983
**Alanine**	0.92	0.70	1.20	0.531	0.983
**Anandamide**	0.87	0.57	1.33	0.511	0.983
**Arachidonic acid**	1.15	0.71	1.86	0.575	0.983
**Arginine**	1.17	0.83	1.65	0.364	0.983
**Asparagine**	1.13	0.73	1.72	0.587	0.983
**Aspartate**	0.98	0.68	1.40	0.897	0.983
**Betaine**	0.96	0.77	1.21	0.747	0.983
**Butyrylcarnitine**	0.98	0.72	1.33	0.906	0.983
**cAMP**	1.00	0.80	1.26	0.973	0.983
**Carnitine**	1.05	0.67	1.64	0.834	0.983
**Choline**	1.31	0.82	2.08	0.253	0.983
**Colchicine**	0.83	0.59	1.17	0.289	0.983
**Creatine**	1.01	0.66	1.54	0.983	0.983
**Cysteamine**	0.88	0.57	1.37	0.576	0.983
**Cysteine**	1.44	0.89	2.33	0.138	0.983
**Cytosine**	1.30	0.89	1.89	0.178	0.983
**DMGV**	1.11	0.83	1.50	0.482	0.983
**GlucosePos2**	0.71	0.45	1.13	0.149	0.983
**Glutamate**	1.07	0.73	1.57	0.736	0.983
**Glutamine**	0.68	0.41	1.11	0.125	0.983
**Histidine**	1.26	0.72	2.19	0.414	0.983
**Isoleucine_Leucine**	0.96	0.65	1.41	0.830	0.983
**Kynurenic acid**	1.22	0.96	1.55	0.102	0.983
**L-Homoserine**	1.21	0.87	1.67	0.261	0.983
**Methionine**	1.04	0.71	1.51	0.844	0.983
**Phenylalanine**	0.97	0.59	1.62	0.921	0.983
**Phosphocholine**	1.17	0.77	1.78	0.460	0.983
**Proline**	1.06	0.72	1.56	0.756	0.983
**Riboflavin**	1.06	0.87	1.28	0.586	0.983
**Serine**	1.07	0.83	1.37	0.620	0.983
**Serotonin**	0.81	0.49	1.33	0.400	0.983
**Spermine**	1.37	0.96	1.95	0.086	0.983
**Taurine**	0.99	0.73	1.36	0.966	0.983

**Thiamine**	1.10	0.76	1.59	0.607	0.983
**Threonine**	0.92	0.59	1.44	0.714	0.983
**Thymidine**	0.90	0.50	1.63	0.734	0.983
**TMAO**	1.03	0.71	1.50	0.880	0.983
**trans-HYP**	0.89	0.71	1.12	0.316	0.983
**Tyrosine**	1.02	0.69	1.51	0.914	0.983
**Uridine**	0.68	0.47	0.98	0.041	0.983
**Valine**	1.28	0.84	1.94	0.257	0.983
**Valine-d8**	1.01	0.75	1.36	0.948	0.983
**1-methylhistamine**	1.05	0.64	1.71	0.850	0.983
**2’-deoxyadenosine**	1.02	0.79	1.31	0.900	0.983
**2-Arachidonyl glycerol**	0.82	0.52	1.27	0.372	0.983
**3-deaazadenosine**	0.78	0.48	1.27	0.323	0.983
**3-IPA**	0.88	0.76	1.03	0.107	0.983
**3HK**	1.12	0.91	1.38	0.300	0.983
**5-Aminolevulinic Acid**	0.98	0.80	1.21	0.883	0.983

Supplementary Table 13. Multivariable Binary Logistic Regression Associations with Presence of Non-Calcified CAD.

Metabolite	Odds Ratio	lower_CI	upper_CI	P value	FDR Adjusted P-value
**α-keto-ß-methylvaleric acid 1**	1.08	0.67	1.73	0.756	0.912
**α-keto-ß-methylvaleric acid 2**	0.92	0.66	1.28	0.606	0.912
**Acetylcarnitine**	0.98	0.79	1.23	0.881	0.960
**Adenosine**	0.94	0.65	1.37	0.757	0.912
**Alanine**	0.96	0.75	1.23	0.751	0.912
**Anandamide**	0.88	0.60	1.29	0.506	0.912
**Arachidonic acid**	0.96	0.62	1.50	0.856	0.960
**Arginine**	0.87	0.63	1.18	0.368	0.880
**Asparagine**	0.98	0.67	1.44	0.929	0.965
**Aspartate**	0.76	0.55	1.06	0.106	0.880
**Betaine**	1.01	0.82	1.24	0.937	0.965
**Butyrylcarnitine**	0.92	0.69	1.22	0.552	0.912
**cAMP**	1.03	0.84	1.26	0.809	0.953
**Carnitine**	1.20	0.80	1.79	0.387	0.880
**Choline**	1.61	1.05	2.49	0.031	0.545
**Colchicine**	0.86	0.63	1.17	0.329	0.880
**Creatine**	1.01	0.69	1.49	0.947	0.965
**Cysteamine**	0.90	0.60	1.33	0.588	0.912
**Cysteine**	0.77	0.50	1.19	0.239	0.880
**Cytosine**	0.86	0.61	1.21	0.399	0.880
**DMGV**	1.05	0.80	1.38	0.717	0.912
**GlucosePos2**	1.09	0.71	1.65	0.703	0.912
**Glutamate**	1.42	1.00	2.03	0.053	0.699
**Glutamine**	0.86	0.56	1.32	0.488	0.912
**Histidine**	0.70	0.42	1.16	0.168	0.880
**Isoleucine_Leucine**	1.17	0.82	1.65	0.386	0.880
**Kynurenic acid**	1.04	0.83	1.29	0.747	0.912
**L-Homoserine**	0.99	0.74	1.33	0.971	0.971
**Methionine**	1.18	0.83	1.67	0.351	0.880
**Phenylalanine**	0.73	0.46	1.15	0.176	0.880
**Phosphocholine**	1.32	0.90	1.94	0.154	0.880
**Proline**	0.93	0.65	1.34	0.700	0.912
**Riboflavin**	1.08	0.91	1.29	0.374	0.880
**Serine**	1.12	0.89	1.41	0.339	0.880
**Serotonin**	0.77	0.49	1.21	0.253	0.880
**Spermine**	1.13	0.82	1.56	0.445	0.912
**Taurine**	0.94	0.70	1.25	0.650	0.912

**Thiamine**	1.13	0.82	1.56	0.457	0.912
**Threonine**	1.10	0.73	1.66	0.646	0.912
**Thymidine**	0.87	0.51	1.50	0.617	0.912
**TMAO**	0.65	0.46	0.92	0.016	0.420
**trans-HYP**	0.98	0.80	1.21	0.871	0.960
**Tyrosine**	1.14	0.80	1.63	0.481	0.912
**Uridine**	0.81	0.58	1.13	0.217	0.880
**Valine**	1.13	0.77	1.66	0.525	0.912
**Valine-d8**	1.14	0.87	1.50	0.347	0.880
**1-methylhistamine**	0.97	0.62	1.51	0.887	0.960
**2’-deoxyadenosine**	1.13	0.90	1.42	0.304	0.880
**2-Arachidonyl glycerol**	1.25	0.83	1.88	0.281	0.880
**3-deaazadenosine**	1.10	0.71	1.70	0.684	0.912
**3-IPA**	0.83	0.72	0.96	0.011	0.420
**3HK**	1.20	0.99	1.45	0.067	0.706
**5-Aminolevulinic Acid**	1.14	0.94	1.37	0.177	0.880

Supplementary Table 14. Multivariable Binary Logistic Regression Associations with Presence of Obstructive CAD.

Metabolite	Odds Ratio	lower_CI	upper_CI	P value	FDR Adjusted P-value
α-keto-ß-methylvaleric acid 1	0.98	0.35	2.73	0.969	0.997
α-keto-ß-methylvaleric acid 2	1.15	0.6	2.19	0.674	0.913
Acetylcarnitine	0.90	0.59	1.38	0.63	0.913
Adenosine	1.39	0.67	2.88	0.373	0.913
Alanine	1.11	0.69	1.8	0.663	0.913
Anandamide	0.62	0.30	1.29	0.203	0.913
Arachidonic acid	1.16	0.48	2.81	0.736	0.913
Arginine	1.08	0.61	1.93	0.793	0.913
Asparagine	1.66	0.81	3.42	0.17	0.913
Aspartate	1	0.55	1.84	0.997	0.997
Betaine	1.18	0.79	1.76	0.431	0.913
Butyrylcarnitine	1.42	0.77	2.62	0.264	0.913
cAMP	1.05	0.74	1.49	0.783	0.913
Carnitine	1	0.42	2.41	0.996	0.997
Choline	1.2	0.51	2.82	0.67	0.913
Colchicine	0.79	0.40	1.55	0.494	0.913
Creatine	1.46	0.67	3.17	0.344	0.913
Cysteamine	0.59	0.27	1.28	0.184	0.913
Cysteine	1.27	0.55	2.95	0.574	0.913
Cytosine	1.25	0.61	2.56	0.534	0.913
DMGV	1.65	0.92	2.96	0.094	0.913
GlucosePos2	0.81	0.37	1.75	0.583	0.913
Glutamate	1.3	0.64	2.66	0.472	0.913
Glutamine	0.89	0.37	2.12	0.787	0.913
Histidine	0.67	0.25	1.75	0.408	0.913
Isoleucine_Leucine	1.2	0.55	2.62	0.651	0.913
Kynurenic acid	1.16	0.74	1.8	0.523	0.913
L-Homoserine	0.95	0.55	1.66	0.867	0.96
Methionine	1.3	0.66	2.56	0.455	0.913
Phenylalanine	1.22	0.51	2.91	0.661	0.913
Phosphocholine	1.91	0.91	4.02	0.087	0.913
Proline	0.89	0.43	1.86	0.756	0.913
Riboflavin	1.24	0.9	1.71	0.183	0.913
Serine	0.96	0.61	1.52	0.87	0.96
Serotonin	0.82	0.38	1.75	0.599	0.913
Spermine	1.3	0.71	2.39	0.39	0.913
Taurine	0.9	0.51	1.57	0.702	0.913

**Thiamine**	1.15	0.68	1.95	0.593	0.913
**Threonine**	0.51	0.23	1.1	0.0857	0.913
**Thymidine**	0.78	0.27	2.25	0.651	0.913
**TMAO**	0.84	0.45	1.59	0.6	0.913
**trans-HYP**	0.92	0.63	1.36	0.684	0.913
**Tyrosine**	1.13	0.54	2.36	0.743	0.913
**Uridine**	0.78	0.43	1.42	0.417	0.913
**Valine**	1.87	0.9	3.91	0.0944	0.913
**Valine-d8**	1.47	0.85	2.53	0.167	0.913
**1-methylhistamine**	0.96	0.41	2.24	0.93	0.997
**2’-deoxyadenosine**	0.99	0.64	1.52	0.962	0.997
**2-Arachidonyl glycerol**	0.79	0.38	1.63	0.516	0.913
**3-deaazadenosine**	0.84	0.38	1.88	0.676	0.913
**3-IPA**	0.72	0.56	0.94	0.016	0.829
**3HK**	1.13	0.8	1.58	0.489	0.913
**5-Aminolevulinic Acid**	0.89	0.63	1.25	0.487	0.913

Supplementary Table 15. Multivariable Linear Regression Associations with Gensini Score.

Metabolite	Beta	lower_CI .2.5 %	upper_CI .97.5 %	P value	FDR Adjusted P-value
α-keto-ß-methylvaleric acid 1	-0.03	-0.29	0.23	0.811	0.896
α-keto-ß-methylvaleric acid 2	-0.17	-0.36	0.02	0.081	0.533
Acetylcarnitine	0.02	-0.11	0.15	0.810	0.896
Adenosine	-0.03	-0.24	0.17	0.747	0.896
Alanine	-0.08	-0.23	0.06	0.247	0.769
Anandamide	-0.01	-0.24	0.22	0.901	0.936
Arachidonic acid	0.11	-0.15	0.37	0.396	0.769
Arginine	0.00	-0.19	0.18	0.960	0.979
Asparagine	-0.16	-0.38	0.06	0.160	0.741
Aspartate	-0.08	-0.27	0.12	0.435	0.769
Betaine	0.04	-0.09	0.16	0.568	0.890
Butyrylcarnitine	0.03	-0.13	0.20	0.705	0.890
cAMP	-0.03	-0.14	0.08	0.584	0.890
Carnitine	0.26	0.03	0.49	0.030	0.402
Choline	-0.03	-0.28	0.23	0.832	0.900
Colchicine	0.04	-0.15	0.22	0.691	0.890
Creatine	0.00	-0.22	0.22	0.997	0.997
Cysteamine	-0.12	-0.34	0.09	0.247	0.769
Cysteine	-0.06	-0.31	0.19	0.636	0.890
Cytosine	0.09	-0.10	0.29	0.346	0.769
DMGV	0.17	0.02	0.32	0.026	0.402
GlucosePos2	0.11	-0.15	0.38	0.396	0.769
Glutamate	0.10	-0.10	0.30	0.333	0.769
Glutamine	0.03	-0.20	0.26	0.801	0.896
Histidine	-0.08	-0.38	0.21	0.572	0.890
Isoleucine_Leucine	-0.04	-0.24	0.16	0.679	0.890
Kynurenic acid	0.11	-0.01	0.23	0.079	0.533
L-Homoserine	0.03	-0.15	0.20	0.772	0.896
Methionine	0.22	0.02	0.43	0.030	0.402
Phenylalanine	0.26	0.01	0.52	0.046	0.402
Phosphocholine	0.02	-0.20	0.23	0.874	0.926
Proline	-0.05	-0.26	0.17	0.674	0.890
Riboflavin	0.12	0.03	0.22	0.014	0.402
Serine	0.06	-0.07	0.20	0.354	0.769
Serotonin	-0.11	-0.37	0.15	0.398	0.769
Spermine	-0.04	-0.22	0.14	0.681	0.890
Taurine	0.05	-0.11	0.22	0.536	0.890

**Thiamine**	0.11	-0.07	0.29	0.242	0.769
**Threonine**	0.12	-0.14	0.37	0.371	0.769
**Thymidine**	-0.17	-0.47	0.14	0.289	0.769
**TMAO**	-0.21	-0.42	-0.01	0.044	0.402
**trans-HYP**	0.03	-0.10	0.15	0.671	0.890
**Tyrosine**	0.15	-0.06	0.36	0.152	0.741
**Uridine**	-0.03	-0.24	0.17	0.752	0.896
**Valine**	-0.05	-0.27	0.17	0.661	0.890
**Valine-d8**	0.06	-0.09	0.22	0.426	0.769
**1-methylhistamine**	-0.17	-0.42	0.08	0.187	0.762
**2’-deoxyadenosine**	0.09	-0.05	0.22	0.202	0.766
**2-Arachidonyl glycerol**	0.12	-0.13	0.37	0.358	0.769
**3-deaazadenosine**	0.18	-0.08	0.43	0.168	0.741
**3-IPA**	-0.07	-0.16	0.01	0.091	0.537
**3HK**	0.05	-0.07	0.16	0.412	0.769
**5-Aminolevulinic Acid**	0.05	-0.06	0.15	0.388	0.769

Supplementary Table 16. Multivariable Linear Regression Associations with CACS.

Metabolite	Beta	lower_CI .2.5 %	upper_CI .97.5 %	P value	FDR Adjusted P-value
**α-keto-ß-methylvaleric acid 1**	-0.20	-0.80	0.40	0.519	0.844
**α-keto-ß-methylvaleric acid 2**	-0.38	-0.82	0.05	0.084	0.422
**Acetylcarnitine**	0.09	-0.21	0.40	0.548	0.844
**Adenosine**	-0.28	-0.75	0.19	0.247	0.595
**Alanine**	-0.21	-0.53	0.12	0.209	0.578
**Anandamide**	0.21	-0.32	0.74	0.434	0.844
**Arachidonic acid**	0.42	-0.16	1.00	0.156	0.516
**Arginine**	0.02	-0.41	0.45	0.922	0.955
**Asparagine**	-0.46	-0.97	0.05	0.076	0.422
**Aspartate**	-0.16	-0.62	0.30	0.494	0.844
**Betaine**	0.19	-0.09	0.48	0.187	0.550
**Butyrylcarnitine**	0.31	-0.08	0.70	0.122	0.453
**cAMP**	-0.01	-0.26	0.25	0.968	0.968
**Carnitine**	0.64	0.10	1.17	0.020	0.263
**Choline**	-0.06	-0.65	0.53	0.829	0.940
**Colchicine**	-0.05	-0.46	0.37	0.829	0.940
**Creatine**	0.23	-0.27	0.73	0.361	0.765
**Cysteamine**	-0.44	-0.95	0.06	0.088	0.422
**Cysteine**	-0.35	-0.92	0.22	0.230	0.581
**Cytosine**	0.02	-0.42	0.47	0.924	0.955
**DMGV**	0.32	-0.03	0.67	0.074	0.422
**GlucosePos2**	0.14	-0.47	0.76	0.645	0.918
**Glutamate**	0.05	-0.43	0.53	0.833	0.940
**Glutamine**	0.11	-0.41	0.64	0.670	0.918
**Histidine**	-0.25	-0.93	0.42	0.458	0.844
**Isoleucine_Leucine**	-0.02	-0.47	0.42	0.921	0.955
**Kynurenic acid**	0.25	-0.03	0.53	0.081	0.422
**L-Homoserine**	-0.08	-0.47	0.31	0.688	0.918
**Methionine**	0.46	-0.01	0.93	0.053	0.422
**Phenylalanine**	0.77	0.19	1.35	0.010	0.263
**Phosphocholine**	-0.02	-0.53	0.48	0.929	0.955
**Proline**	0.09	-0.40	0.59	0.710	0.918
**Riboflavin**	0.27	0.05	0.50	0.018	0.263
**Serine**	-0.05	-0.37	0.26	0.744	0.939
**Serotonin**	-0.09	-0.67	0.50	0.775	0.940
**Spermine**	-0.28	-0.70	0.14	0.184	0.550
**Taurine**	0.29	-0.08	0.67	0.128	0.453

**Thiamine**	0.37	-0.04	0.78	0.077	0.422
**Threonine**	-0.02	-0.62	0.57	0.937	0.955
**Thymidine**	0.21	-0.50	0.92	0.558	0.844
**TMAO**	-0.58	-1.05	-0.10	0.017	0.263
**trans-HYP**	0.09	-0.19	0.38	0.531	0.844
**Tyrosine**	0.40	-0.08	0.88	0.101	0.423
**Uridine**	0.23	-0.23	0.69	0.324	0.716
**Valine**	0.07	-0.45	0.58	0.802	0.940
**Valine-d8**	0.20	-0.15	0.55	0.267	0.615
**1-methylhistamine**	-0.19	-0.79	0.42	0.543	0.844
**2’-deoxyadenosine**	0.19	-0.11	0.50	0.218	0.578
**2-Arachidonyl glycerol**	0.24	-0.34	0.82	0.420	0.844
**3-deaazadenosine**	0.48	-0.10	1.06	0.104	0.423
**3-IPA**	-0.04	-0.24	0.16	0.697	0.918
**3HK**	0.08	-0.18	0.34	0.549	0.844
**5-Aminolevulinic Acid**	0.06	-0.19	0.31	0.626	0.918

Supplementary Table 17. Multivariable Linear Regression Associations with SPS.

Metabolite	Beta	lower_CI .2.5 %	upper_CI .97.5 %	P value	FDR Adjusted P-value
α-keto-ß-methylvaleric acid 1	0.00	-0.28	0.28	0.993	0.993
α-keto-ß-methylvaleric acid 2	0.04	-0.19	0.26	0.752	0.993
Acetylcarnitine	-0.04	-0.19	0.11	0.578	0.993
Adenosine	0.06	-0.17	0.29	0.608	0.993
Alanine	-0.03	-0.20	0.13	0.699	0.993
Anandamide	-0.05	-0.31	0.22	0.728	0.993
Arachidonic acid	0.15	-0.14	0.44	0.318	0.993
Arginine	-0.04	-0.25	0.18	0.743	0.993
Asparagine	-0.18	-0.43	0.06	0.147	0.993
Aspartate	-0.09	-0.31	0.13	0.429	0.993
Betaine	0.06	-0.08	0.20	0.396	0.993
Butyrylcarnitine	0.03	-0.15	0.22	0.727	0.993
cAMP	-0.08	-0.20	0.05	0.244	0.993
Carnitine	0.16	-0.09	0.42	0.211	0.993
Choline	-0.29	-0.58	0.01	0.054	0.993
Colchicine	-0.02	-0.22	0.18	0.843	0.993
Creatine	-0.09	-0.33	0.16	0.486	0.993
Cysteamine	0.09	-0.15	0.33	0.456	0.993
Cysteine	0.02	-0.28	0.31	0.917	0.993
Cytosine	0.05	-0.18	0.28	0.682	0.993
DMGV	0.06	-0.12	0.23	0.523	0.993
GlucosePos2	0.10	-0.19	0.40	0.493	0.993
Glutamate	0.08	-0.16	0.31	0.529	0.993
Glutamine	0.00	-0.26	0.25	0.980	0.993
Histidine	-0.09	-0.43	0.25	0.597	0.993
Isoleucine_Leucine	0.04	-0.18	0.26	0.715	0.993
Kynurenic acid	0.02	-0.12	0.16	0.739	0.993
L-Homoserine	-0.01	-0.20	0.18	0.907	0.993
Methionine	0.14	-0.08	0.37	0.215	0.993
Phenylalanine	0.24	-0.05	0.53	0.106	0.993
Phosphocholine	-0.01	-0.24	0.23	0.961	0.993
Proline	-0.08	-0.32	0.15	0.487	0.993
Riboflavin	0.15	0.04	0.26	0.007	0.367
Serine	0.00	-0.16	0.15	0.981	0.993
Serotonin	0.03	-0.25	0.32	0.813	0.993
Spermine	-0.01	-0.22	0.19	0.891	0.993
Taurine	0.02	-0.17	0.21	0.825	0.993

**Thiamine**	0.17	-0.04	0.37	0.108	0.993
**Threonine**	0.01	-0.28	0.30	0.936	0.993
**Thymidine**	-0.15	-0.49	0.20	0.405	0.993
**TMAO**	-0.09	-0.32	0.15	0.475	0.993
**trans-HYP**	0.00	-0.14	0.15	0.971	0.993
**Tyrosine**	0.10	-0.13	0.34	0.398	0.993
**Uridine**	0.09	-0.14	0.32	0.433	0.993
**Valine**	0.03	-0.22	0.28	0.797	0.993
**Valine-d8**	0.05	-0.12	0.23	0.543	0.993
**1-methylhistamine**	0.01	-0.28	0.30	0.962	0.993
**2’-deoxyadenosine**	0.01	-0.14	0.16	0.891	0.993
**2-Arachidonyl glycerol**	-0.02	-0.31	0.27	0.888	0.993
**3-deaazadenosine**	0.12	-0.18	0.42	0.426	0.993
**3-IPA**	-0.04	-0.14	0.06	0.429	0.993
**3HK**	0.01	-0.12	0.14	0.885	0.993
**5-Aminolevulinic Acid**	-0.01	-0.13	0.11	0.868	0.993

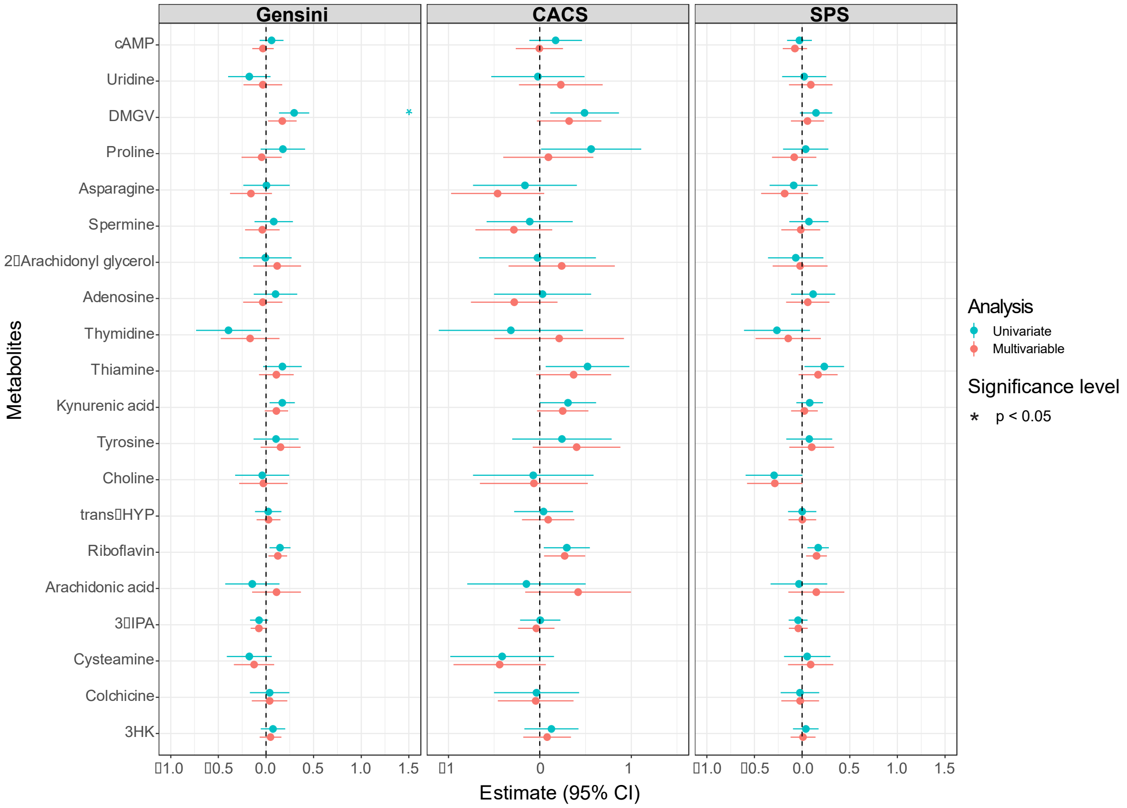

Supplementary Figure 1: Regression coefficients for association of top 20 metabolites for amount of CAD (left), calcified plaque (middle), and soft plaque (right). Ranked according to strength of univariate association with presence of CAD. Dots represent beta coefficients, lines represent 95% confidence intervals. P values are adjusted for a 5% discovery discover rate using Benjamini and Hochberg approach: * p<0.05. Turquoise represents univariate associations. Red represents associations adjusted for age, sex, hypertension, hypercholesterolaemia, diabetes, and significant smoking.
